# Human genetic variations conferring resistance to malaria

**DOI:** 10.1186/s12967-025-07017-w

**Published:** 2025-09-24

**Authors:** Xiaokun Zhang, Jie Wu, Yunxing Peng, Lan Luo, Lu Zhang, Xi Huang, Guoying Chen, Yirong Li, Haoan Yi

**Affiliations:** 1 Functional Laboratory of Experimental Teaching Center, Faculty of Basic Medical Science, Kunming Medical University, Kunming, 650500 Yunnan People’s Republic of China; 2 Emergency Medicine Department of First Affiliated Hospital of Kunming Medical University, Kunming, 650500 Yunnan People’s Republic of China; 3 Department of Cell Biology and Medical Genetics, Faculty of Basic Medical Science, Kunming Medical University, Kunming, 650500 Yunnan People’s Republic of China; 4 Department of Biochemistry and Molecular Biology, Faculty of Basic Medical Science, Kunming Medical University, Kunming, 650500 Yunnan People’s Republic of China

**Keywords:** Malaria resistance, Genetic variations, Hemoglobinopathy, Thalassemia, G6PD deficiency, Evolutionary

## Abstract

Malaria remains one of the most significant public health challenges globally, particularly in tropical and subtropical regions. Throughout evolutionary history, malaria-induced natural selection has profoundly influenced human genetic evolution, leading to the emergence of numerous genetic variations that confer resistance to the disease. These adaptations highlight the complicated interplay between pathogens and human genetics. This review focuses on key genetic variations associated with malaria resistance, including hemoglobinopathies (such as sickle cell trait and thalassemia), glucose-6-phosphate dehydrogenase deficiency, blood group polymorphisms and genetic variants related to inflammation and immune regulation. The prevalence of these genetic adaptations varies widely across different geographic regions, reflecting the historical burden of malaria in those areas. Despite significant advancements in genetic research, the precise mechanisms by which these mutations confer protection against malaria remain incompletely understood. Furthermore, the interactions between these genetic factors and environmental influences add to another layer of complexity. A comprehensive understanding of these genetic variations and their functional implications is crucial for advancing malaria epidemiology, improving diagnostic tools, and developing targeted prevention and control strategies, ultimately contributing to global efforts to eradicate malaria.

**Supplementary Information:**

The online version contains supplementary material available at 10.1186/s12967-025-07017-w.

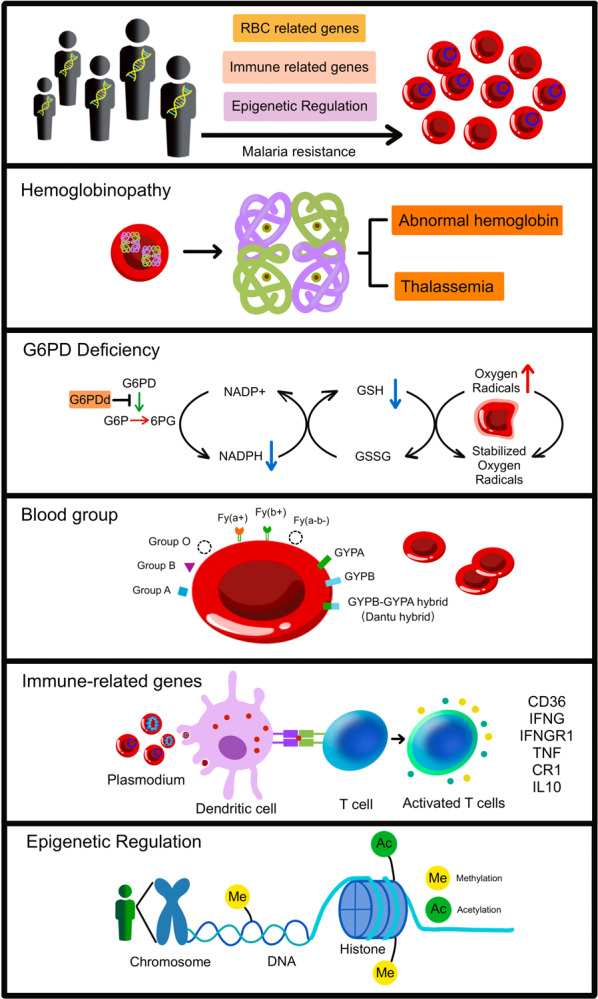

## Introduction

**Fig. 1:**
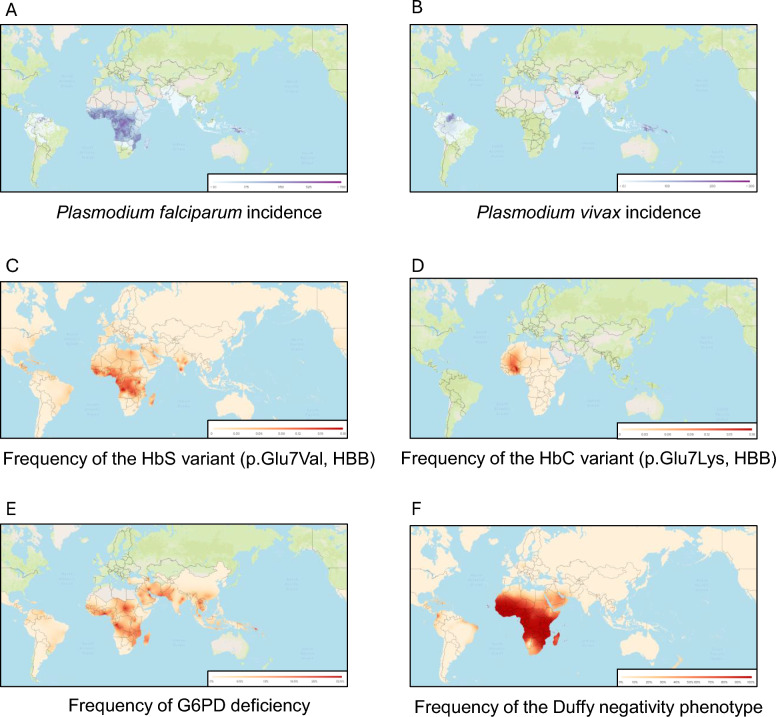
Geographic Patterns of Malaria Incidence and Human Resistance Alleles. The global data on malaria cases and the distribution of selected human genetic traits that reduce malaria risk. Malaria incidence is measured as the number of new cases per 1,000 people each year. Genetic variant frequencies are given as percentages of the population. Data are from the Malaria Atlas Project (https://malariaatlas.org/). Color intensity on the maps reflects either infection rate or gene frequency, with darker colors indicating higher values. **A** *Plasmodium falciparum* incidence. Transmission is highest in Central and Western sub-Saharan Africa and in Papua New Guinea. **B** *Plasmodium vivax* incidence. High rates are observed in Guyana, Pakistan, and Papua New Guinea. In contrast to *P.f*, *P.v* is nearly absent from most of sub-Saharan Africa. **C** Frequency of the HbS variant (p.Glu7Val, HBB). The sickle cell trait (heterozygous HbAS) provides strong protection against severe *P.f* malaria by altering RBC shape and impairing parasite growth. Highest allele frequencies occur in sub-Saharan Africa, consistent with strong historical selection pressure. **D** Frequency of the HbC variant (p.Glu7Lys, HBB). HbC and HbS both affect the same amino acid position (the seventh residue) in the β-globin chain but result in different substitutions. HbC is mainly found in West Africa, especially in Burkina Faso and Ghana. People who inherit two copies (HbCC) have lower parasite levels and partial protection against malaria. Compared to HbS, HbC appeared more recently in human populations and has a more limited geographic distribution. **E** Frequency of G6PD deficiency. G6PD deficiency reduces the capacity of RBCs to generate NADPH, a key molecule for protecting cells against oxidative damage. As a result, affected RBCs are more susceptible to oxidative stress and premature destruction. This deficiency is prevalent in sub-Saharan Africa, the Middle East, South Asia, and Southeast Asia. The increased oxidative stress in infected cells could hinder malaria parasite survival, contributing to partial protection against malaria. **F** Frequency of the Duffy negativity phenotype. The Duffy negativity phenotype results in the absence of Duffy antigen expression on erythrocyte surfaces, which is essential for *P.v* invasion. The Duffy-negative genotype is nearly fixed in populations of West and Central Africa, correlating with the near absence of *P.v* malaria in these regions

Malaria, one of the most prevalent infectious diseases, typically presents with symptoms such as fever, chills, fatigue, vomiting, and headache, and in severe cases may lead to jaundice, seizures, coma, or death [1]. For the majority of the past five millennia, malaria has been a leading cause of premature death in many parts of the world [2]. There are five species of the genus *Plasmodium* that can infect humans: *Plasmodium falciparum* (*P.f*), *Plasmodium malariae* (*P.m*), *Plasmodium ovale* (*P.o*), *Plasmodium vivax* (*P.v*), and *Plasmodium knowlesi* (*P.k*) [3]. The most prevalent species globally are *P.f* and *P.v*, with their distribution varying spatially (Fig. 1A, B). The tropical region has long been the hardest hit by malaria, as populations in these areas are more susceptible to parasite infection due to extensive exposure to Anopheles mosquito bites. According to the World Malaria Report, there were an estimated 597,000 malaria deaths globally, with a mortality rate of 13.7 per 100,000, the African region remained the most affected, accounting for approximately 94% of malaria cases and 95% of malaria deaths worldwide in 2023 [4]. The outbreak of COVID-19 has led to a resurgence of malaria, continuing to pose a significant threat to human health [5, 6].

**Fig. 2:**
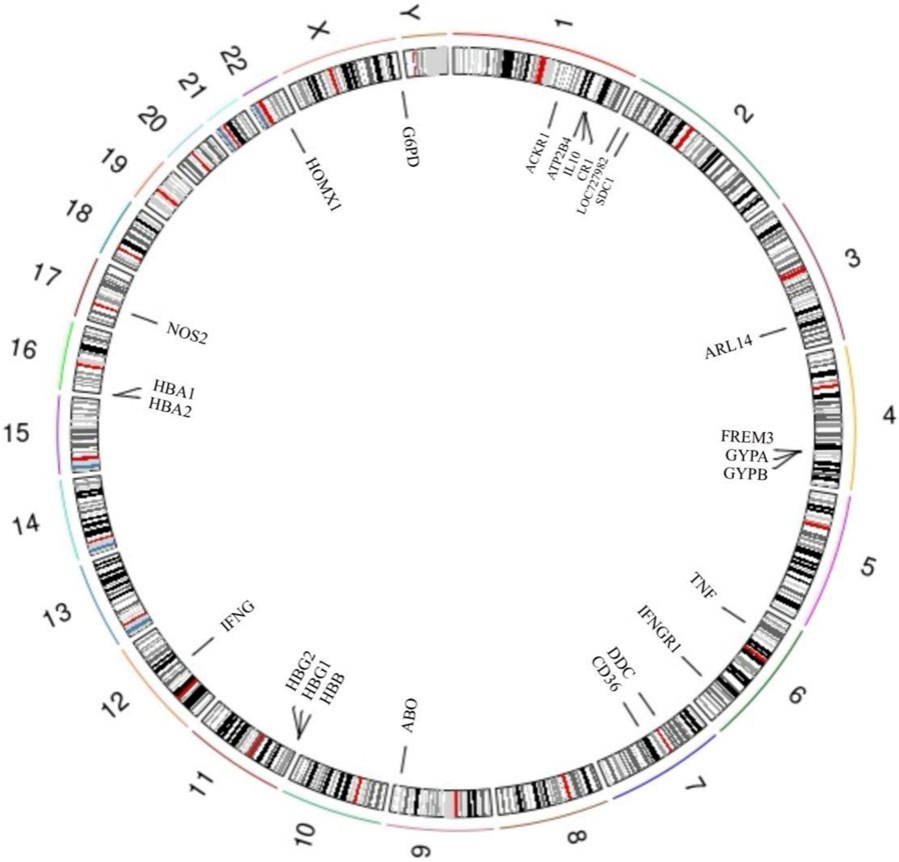
Genomic locations of the genes harboring malaria-protective variants. Chromosomal positions of key human genes containing variantslinked to malaria resistance. These genes encode hemoglobin subunits, RBC membrane proteins, metabolic enzymes, and immune regulators. This genomic map highlights loci under selective pressure due to malaria and provides a framework for understanding host genetic adaptations

*Plasmodium* has coexisted with humans for several millennia, exerting selective pressure on the human genome [7, 8]. The high prevalence of malaria exerts selective pressure, favoring the survival and transmission of specific genetic variations that confer resistance to the disease [9] (Fig. 2). Thus, malaria infection is widely regarded as an evolutionary driver behind human genetic polymorphism and diversity. In this review, we aim to discuss the current evidence of genetic variations that confer resistance to malaria.

## Hemoglobinopathy

Hemoglobin (Hb) is a protein found in red blood cells (RBCs) that plays a vital role in transporting oxygen to tissues throughout the body. Its structure is composed of different combinations of globin chains, and the production of these chains is tightly regulated by genetic mechanisms. The types of hemoglobin vary with developmental stages. During fetal life, the predominant form is fetal hemoglobin (HbF), which consists of two alpha (α) and two gamma (γ) chains (α_2_γ_2_). After birth, HbF is largely replaced by adult forms of hemoglobin. The major type in adults is hemoglobin A (HbA), composed of two alpha and two beta (β) chains (α_2_β_2_), while a smaller proportion is hemoglobin A2 (HbA_2_), composed of two alpha and two delta (δ) chains (α_2_δ_2_).There are two genes for the α and γ chains, and one gene for each of the β and δ chains [10]. Specifically, the *HBA1* (OMIM: 141,800) and *HBA2* (OMIM: 141,850) genes encode the α chain, the *HBB* gene (OMIM: 141,900) encodes the β chain, the *HBD* gene (OMIM: 142,000) encodes the δ chain, and the *HBG2* (OMIM: 142,250) and *HBG1* (OMIM: 142,200) genes encode the γ chain.

Over the long coexistence of malaria and humans, hemoglobin has evolved, resulting in over 3,000 reported variants. Most variants are asymptomatic, but some lead to hemoglobinopathies, which are disorders caused by abnormalities in hemoglobin synthesis or function. These conditions can result in anemia, hemolysis, or abnormalities in blood rheology. Hemoglobinopathies are classified into two major categories: abnormal hemoglobin and thalassemia. Abnormal hemoglobin molecules include Hemoglobin S (HbS), Hemoglobin C (HbC), Hemoglobin E (HbE), among others. Thalassemia is mainly divided into α-thalassemia and β-thalassemia. Since HbA constitutes most of the hemoglobin in adults, abnormalities in either the α or β chains are responsible for most hemoglobinopathies. Hemoglobinopathies affect approximately 7% of the global population, with the highest prevalence in regions such as Africa, the Mediterranean, and Southeast Asia—areas that largely overlap with malaria-endemic zones [11] (Fig. 1C, D), suggesting that malaria has been a crucial factor driving the high frequency of these variations.

### HbS

**Fig. 3:**
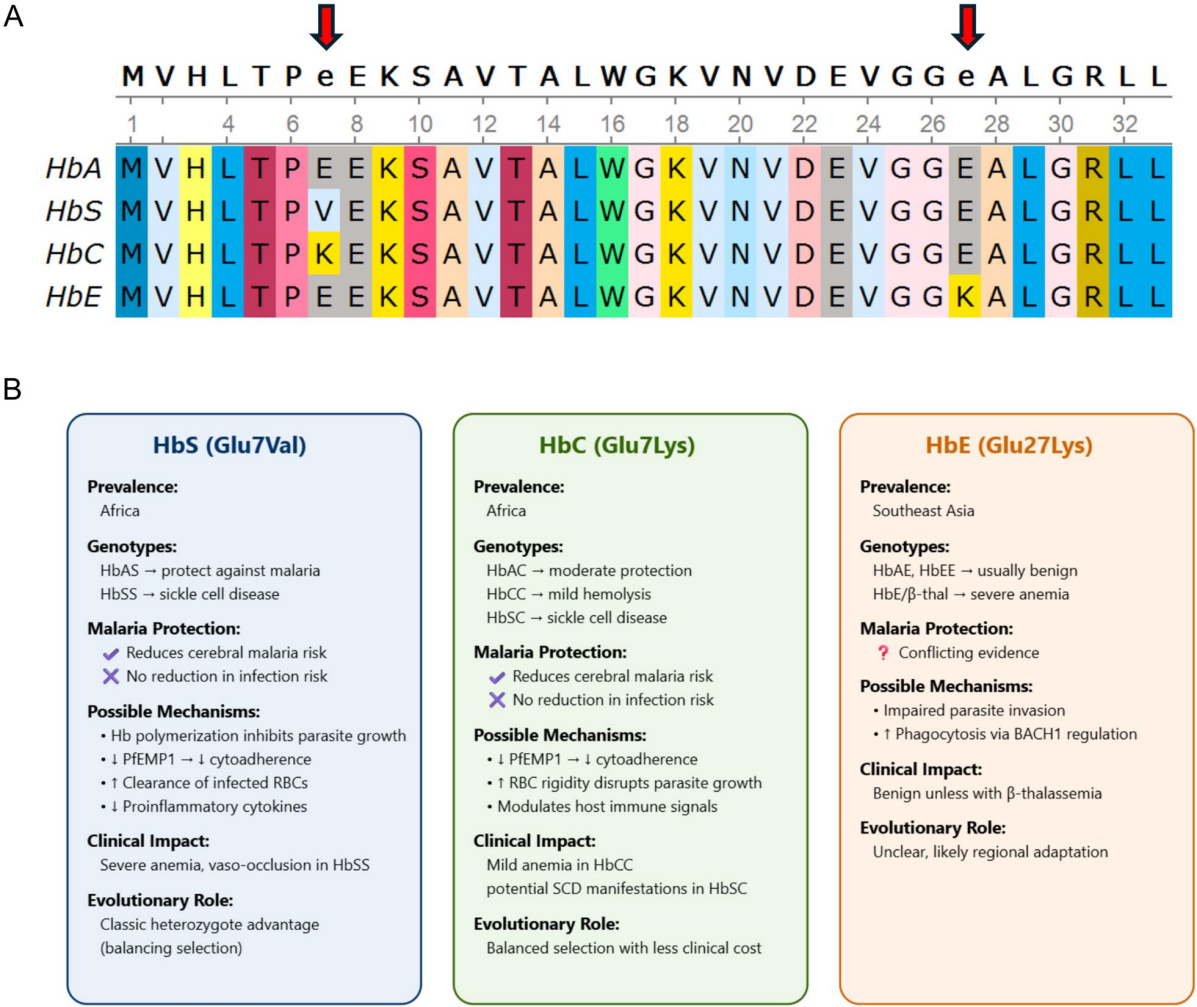
Molecular mutations and functional comparison of abnormal hemoglobin. **A** Molecular mutations of abnormal hemoglobin. Amino acid substitutions in the β-globin chain (*HBB* gene) defining abnormal hemoglobin variants HbS (p.Glu7Val), HbC (p.Glu7Lys), and HbE (p.Glu27Lys), compared to normal adult hemoglobin (HbA). Red arrows indicate the positions of missense mutations, with HbS and HbC affecting the same amino acid residue but resulting in different substitutions. These variants collectively impact hemoglobin polymerization and stability. **B** Functional properties and clinical significance of abnormal hemoglobin. Comparative summary of the geographic distribution, genotypic states, malaria protective effects, underlying mechanisms, clinical manifestations, and evolutionary significance of HbS, HbC, and HbE. HbS and HbC are mainly found in Africa and confer protection against severe *P.f* malaria in heterozygous carriers. Homozygous HbS individuals (HbSS) develop SCD characterized by chronic hemolytic anemia and vaso-occlusive crises. Compound heterozygotes carrying one HbS and one HbC allele (HbSC) typically experience a milder form of SCD with variable severity. Homozygous HbC (HbCC) usually causes a mild hemolytic anemia but is associated with reduced parasite density and partial malaria protection. HbE is prevalent in Southeast Asia, generally causing mild symptoms alone but can cause severe disease when co-inherited with *β*-thalassemia. The protective role of HbE against malaria remains controversial, with conflicting reports in the literature

Hemoglobin S (HbS, *HBB*: p.Glu7Val, OMIM: 141,900.0243) is prevalent in Africa (Figs. 1C, 3A) and causes abnormal molecular structural changes, HbS polymerization, under hypoxic conditions. HbS polymers rapidly elongate to form fibrous strands, inducing erythrocyte sickling [12]. These changes lead to the stiffening and distortion of RBCs, resulting in sickle cell disease (SCD, OMIM: 603,903). SCD affects over 3 million people worldwide, with more than 300,000 infants born annually and an estimated 376,000 deaths in 2021 alone [13–15]. It is one of the leading genetic causes of childhood mortality globally and the 12th leading cause of death in children under five. The burden is particularly severe in sub-Saharan Africa, where 50–90% of affected children die before the age of five due to lack of early diagnosis and adequate care [16], making SCD a major public health challenge in the region. SCD typically presents in infancy with clinical features such as anemia, jaundice, recurrent pain crises, and delayed growth and development. The most severe clinical manifestations occur in individuals with homozygous HbSS or compound heterozygous genotypes such as HbSC and HbS/β^0^-thalassemia. Among these, HbSS is associated with the most serious complications, including frequent vaso-occlusive crises, chronic hemolytic anemia, stroke, acute chest syndrome, and osteonecrosis of the femoral head [15, 17].

The relationship between HbS and malaria provides a classic example of how human genetics can influence susceptibility and resistance to infectious diseases. The protective effect of HbS is primarily against severe malaria, rather than reducing overall infection risk [18–20]. The varying effects of HbS on infection suggest that its protective role against malaria may be related to controlling *P.f* hyperparasitemia [21]. In malaria-endemic regions, HbS exhibits a heterozygote advantage. HbAA individuals are free from SCD but face a higher risk of death from malaria [22], while HbAS individuals are usually asymptomatic and gain protection against severe malaria. Therefore, all three hemoglobin genotypes (HbAA, HbAS, and HbSS) are subject to natural selection. However, their relative fitness varies. In malaria-endemic regions, the heterozygous HbAS genotype exhibits the highest fitness, providing a classic example of balancing selection maintaining genetic polymorphism.

Despite extensive molecular epidemiological studies, the exact mechanisms of HbS resistance to malaria remain unclear. Most studies suggest that HbS hinders parasite growth and reduces parasite density [21]. Initial research posited that HbS enhances the clearance of sickle-infected RBCs [23]. Subsequent studies found that under hypoxic conditions, the oxygen-dependent polymerization of HbS in infected erythrocytes inhibits parasite growth by blocking replication mid-cycle [24]. Furthermore, HbS reduces the adhesion of parasitized RBCs, limiting their sequestration in the brain and other organs, which in turn lowers the incidence of cerebral malaria [25]. This effect may be attributed to HbS decreasing the expression of *P.f*-infected erythrocyte membrane protein 1 (PfEMP1) on the RBC membranes [26]. The downregulation of PfEMP1 expression may be attributed to increased formation of methemoglobin, which disrupts actin remodeling and prevents the parasite from assembling its actin cytoskeletonwithin the host cytoplasm, thereby hindering the transport of PfEMP1 to the erythrocyte surface and ultimately reducing cytoadherence [27–30].

In addition to its effects on RBCs, HbS also regulates the host’s immune response, despite hemoglobin being expressed only in RBCs. In malaria patients with HbS, serum levels of C-X-C motif chemokine ligand 10 (CXCL10), tumor necrosis factor-α (TNF-α), chemokine (C-C motif) ligand 2 (CCL2), interleukin-8 (IL-8), and interleukin-6 (IL-6) are lower compare to malaria patients without HbS [31]. HbS carriers have lower pathogen loads following malaria infection, potentially resulting in reduced antigenic stimulation and a weaker immune response, rather than direct modulation of the immune system by HbS. However, Gerardo Cabrera et al. found that HbS is associated with enhanced immunoglobulin G (IgG) antibody responses to *P.f* variant surface antigens [32]. The mechanisms underlying this apparent immunological paradox remain unclear. It is uncertain whether these effects result from direct modulation of immune pathways or are secondary to altered parasitemia. Further research is needed to clarify how HbS shapes the balance between immune protection and immunopathology.

In summary, HbS confers adaptive protection against severe malaria, shaped by malaria-driven selective pressure. The heterozygous genotype (HbAS) exhibits a fitness advantage by balancing malaria resistance with avoidance of sickle cell disease pathology. Mechanistically, HbS protection involves parasite growth inhibition, decreased cytoadherence, and immune modulation, though precise pathways remain to be fully elucidated.

### HbC

Hemoglobin C (HbC, *HBB*: p.Glu7Lys, OMIM: 141,900.0038) occurs at high frequencies in West Africa (Figs. 1D, 3A). HbC heterozygotes (HbAC) are typically asymptomatic, while HbC homozygotes (HbCC) experience mild hemolytic anemia due to decreased RBCs solubility and crystal formation [33]. Both HbC and HbS are point mutations affecting the same amino acid residue in the β-globin chain encoded by the *HBB* gene, where glutamic acid is substituted by lysine in HbC and by valine in HbS. HbC can occur independently, with HbA, or as part of the double variant HbCC. Generally, the risk and severity of SCD associated with HbC are milder compared to those caused by HbS. However, A 20 year’s longitudinal follow-up of a Jamaican birth cohort demonstrated that individuals with HbSC disease have a significantly higher risk of developing proliferative sickle cell retinopathy compared to those with HbSS [34]. Furthermore, viral infections like COVID-19 or dengue may synergize with HbSC-specific pathophysiological traits, such as higher viscosity, susceptibility to bone marrow necrosis fat embolism syndrome, and altered immune response, resulting in unexpectedly severe clinical outcomes [35]. These findings challenge the traditional perception of HbSC as a benign or milder variant of SCD.

Similar to HbS, HbC reduces the risk of severe malaria but does not lower the overall risk of *Plasmodium* infection [36, 37]. Numerous studies report that HbC inhibits malaria parasite proliferation in vivo [38, 39]. The resistance mechanism of HbC to malaria is similar to that of HbS and can be divided into three aspects: (a) it affects the host’s immune response [40], (b) it impacts the growth and reproduction of *Plasmodium* [38], and (c) it disrupts actin, affecting the expression of *P.f* proteins in erythrocyte membranes [41]. These shared protective effects may stem from the fact that both HbS and HbC arise from point mutations at the same amino acid residue in the β-globin chain. In both homozygous (HbCC) and double heterozygous (HbSC) states, the protective effects against malaria surpass those in HbAS individuals [42].

In summary, HbAS offers significant protection against severe malaria, with limited effect on infection rates. HbSC, being a compound heterozygote, combines the benefits of HbS and HbC and likely provide stronger protection than either HbAS or HbAC, though at the cost of potential SCD manifestations.

### HbE

Hemoglobin E (HbE, *HBB*: p.Glu27Lys, OMIM: 141,900.0071) is an abnormal hemoglobin that typically does not cause significant pathological changes in RBCs, and carriers generally do not exhibit obvious clinical symptoms (Fig. 3A). HbE is prevalent throughout Southeast Asia, including eastern India, northern Thailand, Cambodia, and Myanmar, with prevalence rates as high as 60% in some regions [43, 44]. Regions where HbE is endemic also have high rates of β-thalassemia, leading to frequent coinheritance of HbE and β-thalassemia, known as HbE/β-thalassemia [45]. Despite HbE typically not causing significant clinical symptoms, HbE/β-thalassemia represents the most prevalent severe form of β-thalassemia in Asia, comprising approximately 50% of clinically severe β-thalassemia disorders globally [46]. HbE/β-thalassemia represents a major public health burden in Southeast Asia [47], up to half of patients present with severe phenotypes requiring life-long transfusions, resulting in complications from iron overload and impaired quality of life [48].

The protective role of HbE against malaria remains controversial and requires further research for confirmation. Early studies found that HbE lacks a selective advantage in protecting against *P.f* infection [49]. A case–control study in Bangladesh indicated that HbE homozygotes (HbEE) are associated with an increased incidence of *P.f* [50]. Another observational study found that HbE did not affect *P.v* parasitemia and the density of *P.f* significantly increased in heterozygous HbE (HbEA) patients [51]. Similarly, other small cohort studies have found no protective advantage of HbE against malaria infection [52, 53]. However, a community survey supported the protective effect of HbE against asymptomatic *P.f* and *P.v* infections [54]. Our previous research on the China-Myanmar border found that HbE reduced the risk of *P.v* infection and parasite density in the Kachin ethnic group [55]. Additionally, a case-control study suggested that HbE only has a protective effect when considering fava bean consumption as a source of oxidative stress, indicating that the resistance of HbE to malaria may be diet-related [56].

In vitro studies suggest that HbE may provide some resistance to *Plasmodium* infection. Chotivanich et al. demonstrated that HbAE RBCs exhibit a membrane abnormality that allows most RBCs to resist invasion by *P.f* [57]. Kanitta Srinoun et al. proposed that HbE RBCs can augment the phagocytic activity of monocytes by modulating the regulation of BTB domain and CNC homolog 1(BACH1), leading to increased *Plasmodium* antigen exposure and immune clearance [58].

Abnormal hemoglobin HbS, HbC and HbE represent distinct missense mutations in the β-globin gene, each arising under selective pressure from malaria but exhibiting divergent biochemical properties, geographic distributions, and clinical outcomes (Fig. 3B). HbS is highly prevalent in sub-Saharan Africa and is the most extensively studied due to its well-characterized role in SCD. In heterozygous carriers (HbAS), HbS confers strong protection against severe *P.f* malaria through multiple RBC–intrinsic mechanisms, including impaired cytoadherence, altered parasite protein trafficking, and enhanced phagocytic clearance of parasitized erythrocytes. HbC predominantly found in West Africa, is also associated with reduced risk of severe malaria, though its protective effect is comparatively moderate. HbC homozygosity results in mild hemolytic anemia and reduced surface expression of PfEMP1, limiting parasite adhesion. In contrast, HbE prevalent in Southeast Asia, is typically clinicallysilent but can cause β-thalassemia syndromes when co-inherited with β^0^ or β⁺ alleles. Although the protective role of HbE against malaria remains controversial, some studies report reduced *P.v* parasite density and enhanced immune clearance in carriers, possibly via altered erythrocyte membrane structure or increased phagocytosis. From an evolutionary perspective, HbS and HbC likely rose to high frequencies under selective pressure from *P.f*, the dominant and most lethal malaria species in Africa. In contrast, the high prevalence of HbE in regions historically endemic for *P.v* suggests it may have evolved under pressure from this species, particularly in parts of Southeast Asia (Supplement Fig. 1A). Together, these hemoglobinopathies illustrate how different *Plasmodium* species have shaped human genetic diversity through region-specific natural selection, leading to distinct erythrocyte phenotypes with variable effects on parasite invasion, replication, and disease severity.

### β-Thalassemia

**Fig. 4:**
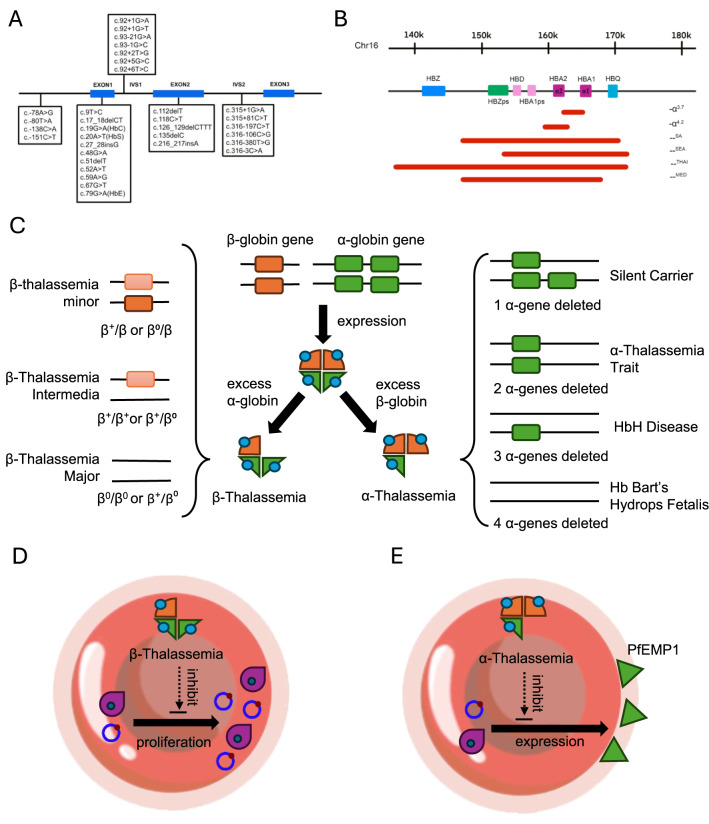
Genetic mutations, clinical phenotypes, and malaria resistance mechanisms of thalassemia. **A** Mutations in the *HBB* gene causing β-thalassemia and abnormal hemoglobin. The human β-globin gene (*HBB*) harbors a diverse set of mutations that either impair β-globin synthesis or alter hemoglobin structure. These include β-thalassemia mutations—typically point mutations, small insertions/deletions, or splicing defects—that reduce (β⁺) or abolish (β^0^) β-globin production, leading to globin chain imbalance, ineffective erythropoiesis, and chronic hemolytic anemia. In parallel, structural variants such as HbS (p.Glu7Val), HbC (p.Glu7Lys), and HbE (p.Glu27Lys) result from amino acid substitutions in exon 1 or exon 2 and produce abnormal hemoglobin molecules that affect RBC deformability and stability. Although clinically deleterious in homozygous or compound heterozygous states, both β-thalassemia and abnormal hemoglobins are maintained at high frequencies in malaria-endemic regions due to their protective effects, likely mediated by impaired parasite growth, enhanced clearance of infected cells, or reduced cytoadherence. **B** Deletional variants in the α-globin gene cluster. The α-globin gene cluster, located on chromosome 16, contains two functional genes (*HBA1* and *HBA2*) as well as pseudogenes and regulatory elements. Deletions within this cluster are the primary cause of α-thalassemia and vary in size and severity. Single-gene deletions, such as − α^3.7^ and − α^4.2^, result in α⁺-thalassemia by removing one copy of *HBA1* or *HBA2*. Larger deletions, including –^SEA^, –^SA^, –^THAI^, and –^MED^, remove both α-globin genes along with adjacent sequences, leading to α^0^-thalassemia. The extent of gene loss correlates with clinical severity, ranging from silent carriers to lethal hydrops fetalis. **C** Classification of α- and β-thalassemia syndromes. Thalassemia syndromes are classified based on the number and type of mutations affecting α- or β-globin genes, which result in an imbalance between α- and β-globin chain production. In β-thalassemia, reduced or absent β-globin synthesis leads to accumulation of unpaired α-globin chains, causing ineffective erythropoiesis and hemolytic anemia. Clinically, β-thalassemia is categorized into silent carrier (one mild mutation), thalassemia intermedia (compound heterozygous or mild mutations), and thalassemia major (homozygous or severe mutations), with increasing severity of anemia and transfusion dependence. In α-thalassemia, disease severity is determined by the number of deleted or dysfunctional α-globin genes. Deletion of one gene result in a silent carrier state; deletion of two genes causes α-thalassemia trait, which is usually asymptomatic or mildly anemic. Deletion of three genes leads to HbH disease, characterized by the formation of β_4_ tetramers and moderate to severe anemia. Deletion of all four α-globin genes results in Hemoglobin Bart’s hydrops fetalis, a lethal condition marked by γ_4_ tetramer formation, severe fetal hypoxia, and intrauterine death. **D** Inhibition of parasite proliferation in β-thalassemia. Although β-thalassemia does not prevent invasion of erythrocytes by parasite, it confers protection by restricting parasite growth and survival within infected RBCs. This effect is mediated by altered erythrocyte phosphorylation patterns, increased oxidative stress, impaired hemozoin formation, and enhanced phagocytic clearance of parasitized erythrocytes. Together, these mechanisms reduce parasite density and delay the progression of severe malaria. **E** Reduced PfEMP1 expression in α-thalassemia. RBCs from individuals with α-thalassemia exhibit decreased surface expression of *P.f* erythrocyte membrane protein 1 (PfEMP1), a critical adhesin mediating cytoadherence of infected erythrocytes to vascular endothelium. This reduction impairs microvascular sequestration and limits inflammatory responses, thereby contributing to protection against severe malaria manifestations

β-thalassemia (OMIM: 613,985) is caused by point mutations mainly in the *HBB* gene, resulting in decreased β-globin chain production. Approximately 1.5% of the global population are carriers of β-thalassemia, with higher frequencies in the Mediterranean region, the Middle East, Central Asia, Southeast Asia and North America [59]. Southeast Asia accounts for about 50% of the world’s carriers of β-thalassemia [60]. The β-globin gene comprises three exons and two intervening sequences (IVS). Over 300 types of β-thalassemia mutations have been identified worldwide, but more than 90% of cases are caused by around 40 of these mutations (Fig. 4A). Based on genetic effects, mutations can be classified into three categories: (a) β^0^ mutations, resulting in no β-globin chain production; (b) β^+^ mutations, reducing the rate of β-globin chain synthesis; (c) β mutations, also known as silent β-thalassemia mutations.

β-thalassemia is clinically classified into three major forms, minor, intermedia, and major, based on the severity of β-globin chain synthesis impairment, clinical manifestations, and transfusion requirements. β-thalassemia minor typically results from heterozygous mutations such as β^+^/β or β^0^/β. Affected individuals are generally asymptomatic or present with only mild microcytic, hypochromic anemia. Hemoglobin A_2_ (HbA_2_) levels are usually elevated (> 3.5%), and no treatment is required [61]. However, genetic counseling is essential, as offspring may inherit more severe forms if both parents are carriers. β-thalassemia intermedia arises from homozygous or compound heterozygous mutations (β^+^/β^+^ or β^+^/β^0^). Its clinical severity is often modulated by genetic modifiers, such as the coinheritance of α-thalassemia. If the patient also inherits α-thalassemia, the resulting reduction in α-globin chain production can alleviate clinical symptoms by mitigating the imbalance between α- and β-globin chains. The clinical phenotype is variable and typically includes moderate anemia (hemoglobin 6–9 g/dL), splenomegaly, and occasional skeletal deformities. Although regular transfusions may not be required, patients are still at risk of developing complications such as iron overload, thromboembolic events, and pulmonary hypertension [62, 63]. β-thalassemia major (Cooley’s anemia) represents the most severe form and is usually caused by β^0^/β^0^ or severe β^+^/β^0^ genotypes. Affected infants typically present within the first year of life with profound microcytic, hypochromic anemia, hepatosplenomegaly, growth retardation, and characteristic craniofacial features such as frontal bossing and maxillary prominence [64]. In the absence of appropriate treatment, these patients experience marked growth impairment and significantly reduced life expectancy [65]. The most frequent causes of death include cardiac complications, particularly congestive heart failure and arrhythmias, largely attributable to chronic iron overload resulting from long-term transfusion therapy [66]. Iron chelation and transfusion remain standard treatments but arelimited by efficacy, access, and long-term safety (Fig. 4C).

However, although β-thalassemia is highly prevalent in malaria-endemic regions, including the Mediterranean basin, the Middle East, South and Southeast Asia, the molecular epidemiological evidence supporting its role in conferring resistance to malaria remains limited (Table 1). A population-based study in Senegal found that three *HBB* variants, rs7946748, rs334 (HbS), and rs713040 (Hemoglobin OKAYAMA), were significantly associated with protection against severe malaria [67]. Notably, in many regions the carrier frequency of β-thalassemia is relatively low, which poses a significant challenge for assembling adequately powered case-control cohorts. This limitation often hampers the ability to detect statistically robust associations between β-thalassemia and malaria-related outcomes, thereby contributing to the scarcity of conclusive epidemiological evidence. A limited number of studies have nonetheless elucidated potential mechanisms by which β-thalassemia may confer resistance to malaria. Early studies indicated that it does not significantly inhibit the invasion of *P.f* [68], suggesting that its protective effect may not stem from preventing parasite entry into erythrocytes. Subsequent research has shown that individuals with the β-thalassemia trait tend to have lower average *P.f* parasite densities compared to those with normal hemoglobin (HbAA) [69]. Heterozygous β-thalassemia results in significant protein phosphorylation changes in parasitized erythrocytes, accompanied by varying degrees of hemolysis and parasite growth inhibition [70]. These findings suggest that β-thalassemia confers a degree of resistance to malaria by limiting parasite replication and survival, rather than by blocking initial invasion (Fig. 4D).

**Table 1:** Summary of the *HBB* gene variants with malaria resistance

Genetics change	Protein change	dbSNP	Reported region	Alter Allele frequency					References
				African	East Asian	South Asian	American	European	
c.9C > A(Hemoglobin OKAYAMA)	p.His3Gln	rs713040	American, Western Africa	0.8710	0.4978	0.6472	0.9715	0.8446	[67]
c.19G > A(HbC)	p.Glu7Lys	rs33930165	Africa	0.01452	0.0000	0.00001098	0.0003832	0.00001104	[220–223]
c.20A > T(HbS)	p.Glu7Val	rs334	African, America	0.04949	0.0000	0.0008897	0.003316	0.0000	[19, 25, 220, 224]
c.79G > A(HbE)	p.Glu27Lys	rs33950507	South Asian, East Asian	0.00006664	0.0008469	0.002580	0.00003332	0.000006782	[58, 225–227]
c.315 + 81C > T^*^	/	rs7946748	Western Africa	0.0651	0.0248	0.1970	0.0840	0.1342	[67]

Allele frequency data for most variants are derived from gnomAD v4—Exomes, reported for African, East Asian, South Asian, American, and European populations

*The allele frequency for variant c.315 + 81C > T is obtained from the 1000 Genomes Project. References correspond to studies investigating these variants and their roles in malaria protection

However, the mechanism underlying β-thalassemia–mediated inhibition of growth and replication remains poorly understood. Martiney et al. discovered that zinc protoporphyrin IX (ZnPP), a heme analog, inhibits the formation of hemozoin in *Plasmodium* trophozoite extracts. Elevated ZnPP levels in erythrocytes with the β-thalassemia trait may partially explain the resistance to malaria [71]. Luzzi et al. observed significantly enhanced phagocytosis of infected β-thalassemic erythrocytes compared to normal infected erythrocytes [72]. Ayi et al. further analyzed the underlying mechanism, finding higher levels of membrane-bound hemichromes, autologous IgG, complement C3c fragments, and aggregated band 3 in β-thalassemic RBCs [73]. Föller et al. suggested that β-thalassemia accelerates RBCs apoptosis, and that premature apoptosis of infected RBCs may facilitate the clearance of parasitized cells and delay the progression of parasitemia [74]. Collectively, these findings suggest that β-thalassemia may confer partial protection against malaria through multiple red cell–intrinsic mechanisms, including impaired hemozoin formation, enhanced immune-mediated clearance of infected erythrocytes, and increased susceptibility of parasitized cells to premature apoptosis.

### α-Thalassemia

α-thalassemia (OMIM: 604,131) results from reduced or absent production of α-globin chains and is prevalent in Mediterranean region, Middle East, Indian subcontinent, and Southeast Asia, with carrier rates reaching up to 80% [75, 76]. Clinical manifestations include anemia, splenomegaly, jaundice, growth retardation, and skeletal changes. The α-globin chains are coded by *HBA1* and *HBA2*.

The most common α-thalassemia genotypes worldwide include the single-gene deletions –α^3.7^ and –α^4.2^, as well as the double-gene deletions –^SA^, –^SEA^, –^MED^, and –^Thai^. The –α^3.7^ deletion is the most prevalent α-thalassemia allele globally, with high frequencies observed throughout Africa, the Mediterranean, the Middle East, and large parts of Asia. The –α^4.2^ deletion is predominantly observed in Southeast Asia and southern China. The –^SA^, –^SEA^, and –^THAI^ alleles are mainly confined to Southeast Asia and southern China, whereas the –^MED^ deletion is primarily found in the Mediterranean basin and the Middle East (Table 2, Fig. 4B). The severity of α-thalassemia is determined by the number of abnormal α-globin genes: (a) Silent carrier and trait α-thalassemia (1 and 2 α-gene deletions) generally show no clinical symptoms; silent carriers have an α/β chain synthesis ratio of 0.9, while trait patients have a ratio of 0.6 with microcytic hypochromic RBCs and occasional hemoglobin H (HbH) inclusions; (b) HbH disease (3 α-gene deletions) presents with a synthesis ratio of 0.3 ~ 0.6, leading to mild to moderate anemia, hepatosplenomegaly, jaundice, and significant hypochromic RBCs with 5% ~ 40% HbH on electrophoresis; (c) Hb Bart’s hydrops fetalis syndrome (4 α-gene deletions) is the most severe form, characterized by complete α-chain absence, high Hb Bart levels (80% ~ 100%), severe tissue hypoxia, and typically results in intrauterine or early neonatal death (Fig. 4C).

**Table 2:** Summary of common α-thalassemia mutations

Mutation	Deletion bp(kb)	Delete gene	Reported region	References
-α^3.7^	3.7	HBA1	Mediterranean,Southeast Asia,Middle East,African,Eastern Asia	[201, 225, 228–231]
-α^4.2^	4.2	HBA2	Southeast Asia,South China	
–^SA^	23	HBZps, HBD, HBA1ps, HBA1, HBA2, HBQ	Southeast Asia	
–^SEA^	20	HBD, HBA1ps, HBA2, HBA1, HBQ	Southeast Asia,South China	
–^THAI^	33.5	HBZ, HBZps, HBA1ps, HBA2, HBA1, HBQ	Southeast Asia,South China	
–^MED^	18	HBZps, HBA1ps, HBA2, HBA1	Mediterranean,Middle East	

Although α-thalassemia has a certain global distribution, few molecular epidemiological studies have provided conclusive evidence that it confers resistance to malaria. Early research suggested that α-thalassemia could reduce the risk of malaria infection [77]. However, subsequent studies found no direct link between α-thalassemia and the risk of *P.f* infection or parasite density but identified a significant negative correlation with the incidence of severe malaria and anemia [78, 79]. Further in vitro studies have demonstrated that parasitized α-thalassemia RBCs exhibit altered and often reduced surface expression of PfEMP1, leading to impaired cytoadherence to endothelial, RBCs and immune cells [72, 80]. Udomsangpetch et al. further demonstrated that *P.f*-infected α-thalassemic RBCs exhibit significantly reduced cytoadherence as well as diminished rosetting formation, suggesting multiple adhesion-related mechanisms underlying malaria protection [81] (Fig. 4E). This resembles the malaria resistance mechanisms reported for HbS and HbC [82]. It should be noted that the presence of HbS and α-thalassemia individually provides protection against severe *P.f* malaria. However, when both traits are inherited together, this protective effect against malaria is almost entirely lost, which may also explain why α-thalassemia has not become fixed in any population in sub-Saharan Africa [83]. Notably, patients who carry both α-thalassemia and β-thalassemia experience alleviation of clinical symptoms due to the mitigation of globin chain imbalance, suggesting that selective interactions exist between α- and β-thalassemia. This interplay also affects the statistical power of studies assessing their protective effects against malaria.

### Fetal hemoglobin

Fetal hemoglobin (HbF), composed of α_2_γ_2_ chains, is typically expressed only in fetuses. The *HBG1* and *HBG2* genes, which encode the γ chains of HbF, are usually suppressed shortly after birth, allowing the expression of *HBB* and *HBD*, which form adult hemoglobin. Studies have found that increased HbF levels are associated with reduced malaria risk [84], but G. Pasvol et.al confirmed through studies using mixtures of HbF and HbA dominant RBCs that *P.f* invasion is not affected by the presence of HbF, but rather by the age of the RBCs [85], possibly due to HbF stability or effects on cell adhesion [86]. During the first few months of life, HbF and maternally derived IgG are thought to act synergistically to reduce the cytoadherence of parasitized RBCs, thereby contributing to protection against malaria [87]. HbF inhibits HbS polymerization and is linked to increased *P.f* proliferation in HbSS RBCs exhibiting higher mean corpuscular fetal hemoglobin levels [88], highlighting the complex molecular interplay between SCD and malaria. Overall, theevidence supporting the effect of HbF on parasite growth remains inconclusive. Within the context of polygenic effects in malaria protection, HbF may play either a beneficial or detrimental role.

## Glucose-6-phosphate dehydrogenase deficiency

**Fig. 5:**
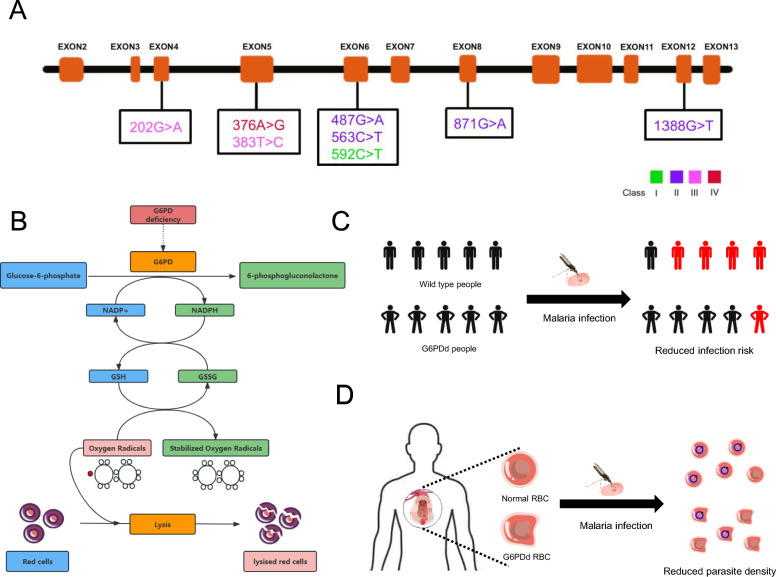
G6PD gene mutations and mechanisms of malaria protection. **A** Classification and positional distribution of G6PD variants associated with malaria protection. Representative G6PD variants are mapped along the gene structure and classified according to enzymatic activity and clinical severity. Variants fall into four World Health Organization classes (I ~ IV), with Class II and Class III predominating in malaria-endemic populations. Protective variants tend to cluster in regions critical for enzyme function, conferring a selective advantage by modulating redox balance in erythrocytes. **B** Role of G6PD in redox homeostasis. G6PD catalyzes the first step of the pentose phosphate pathway, converting glucose-6-phosphate into 6-phosphogluconolactone while generating NADPH. NADPH is essential for maintaining reduced glutathione levels, which detoxify reactive oxygen species (ROS) and protect erythrocytes from oxidative damage. In G6PD deficiency, impaired NADPH production compromises antioxidant defense, resulting in increased susceptibility of RBCs to oxidative stress and hemolysis. **C** G6PD deficiency reduces malaria infection risk. Epidemiological studies consistently demonstrate that G6PDd is associated with a decreased risk of *P.f* and *P.v* infection in endemic populations. The altered redox environment in G6PDd erythrocytes is thought to impair parasite invasion, contributing to lower infection prevalence and clinical incidence. **D** G6PD deficiency limits parasite proliferation. In G6PDd erythrocytes, increased oxidative stress and impaired redox balance compromise *Plasmodium* survival and growth. Enhanced fragility of infected RBCs facilitates their premature clearance by phagocytes, resulting in reduced parasite density and mitigating the severity of malaria

Glucose-6-Phosphate Dehydrogenase Deficiency (G6PDd, OMIM: 300,908) is an X-linked incomplete dominant disorder primarily found in Southeast Asia and Africa (Fig. 1E). G6PDd impairs the pentose phosphate pathway, leading to diminished production of nicotinamide adenine dinucleotide phosphate (NADPH) in RBCs. As NADPH is essential for the regeneration of reduced glutathione (GSH), a critical antioxidant that neutralizes reactive oxygen species (ROS), G6PDd erythrocytes exhibit heightened vulnerability to oxidative stress. Upon exposure to oxidizing agents, such as infections, certain drugs (e.g., sulfonamides, antimalarials), or fava bean ingestion, intracellular ROS accumulate, leading to oxidative damage of hemoglobin and the RBCs membrane. This process results in the formation of Heinz bodies, membrane instability, and ultimately intravascular or extravascular hemolysis (Fig. 5B).

There are up to 217 described mutations in the *G6PD* gene, each affecting the enzyme stability and catalytic efficiency to varying degrees [89]. The WHO classifies G6PDd variants into four severity classes [90]: I. Severe mutations, resulting in a total loss of normal G6PD function; II. Intermediate mutations, causing more than 90% loss of normal G6PD function; III. Mild mutations, leading to a 40–90% loss of normal G6PD function; IV. Asymptomatic variants, with 0–40% loss of normal G6PD function. The clinical manifestations of *G6PD* variants range from very mild, with almost no symptoms, to severe acute hemolytic anemia (AHA). Most individuals with G6PDd are asymptomatic and have a normal lifespan. In countries where G6PDd is prevalent, it is likely the most common cause of neonatal jaundice and is associated with increased risks of acute kidney injury, severe infections, transfusion-dependent anemia, early mortality, and heightened susceptibility to Gram-positive bacterial infections [91–93]. Severe *G6PD* variants can lead to chronic non-spherocytic hemolytic anemia, a rare condition marked by lifelong hemolysis, intermittent exacerbations, and complications such as cholelithiasis [94].

The distribution of G6PDd largely coincides with malaria-endemic regions, and many common mutations have been demonstrated to provide resistance against malaria (Table 3, Fig. 5A). The prevalence of G6PDd is well established as a result of natural selection for malaria resistance. The distribution of different *G6PD* variants in relation to *P.f* and *P.v* infections shows that 202 G > A and 376 A > G variants are likely under stronger selective pressure from* P.f*, while 487 G > A, 563 C > T, 592 C > T, 871 G > A, and 1388 G > A variants appear to be more influenced by selection from *P.v* (Supplement Fig. 1B). Numerous studies have confirmed that G6PDd reduces the risk of *P.f* infection [95, 96]. Our previous case–control studies in the China-Myanmar border region indicated that the *G6PD* Mahidol 487G > A mutation also lowers the risk of *P.v* infection [97]. Research has shown that malaria parasites survive poorly in G6PDd blood [98, 99]. Further analysis of blood G6PD activity revealed that individuals without *Plasmodium* infection had lower peripheral blood G6PD activity compared to malaria patients [100] (Fig. 5C, D). However, some studies found no significant reduction in malaria parasite density in the G6PDd group compared to the normal group [101, 102]. It is important to note that the *G6PD* gene is located on the X chromosome, G6PD activity can be affected by random X-inactivation in females [103]. This may introduce errors and biases in analyzing G6PD activity among heterozygous females and its impact on malaria resistance.

**Table 3:** Summary of the *G6PD* gene variants with malaria resistance

Mutation	Genetics change	Protein change	Exon	Class	dbSNP	Reported region	Alter Allele Frequency					References
							African	East Asian	South Asian	American	European	
G6PD A-	202 G > A	Val68Met	4	III	rs1050828	Africa, American	0.1228	0.000	0.0002990	0.006565	0.000	[67, 95, 96, 99, 232]
G6PD A-	376 A > G	Asn126Asp	5	IV	rs1050829	African, American	0.3188	0.000	0.0006156	0.02174	0.0004022	[95, 96, 233]
G6PD Vanua Lava	383 T > C	Leu128Arg	5	III	rs78365220	Mediterranean regions	0.000	0.000	0.000	0.000	0.00001228	[234]
G6PD Mahidol	487 G > A	Gly163Ser	6	II	rs137852314	Southeast Asians, East Asian	0.000	0.0001482	0.0005096	0.000	0.000	[97, 235]
G6PD Mediterranean	563 C > T	Ser188Phe	6	II	rs5030868	South Asia	0.0002097	0.000	0.01867	0.00002181	0.0001900	[236]
G6PD Coimbra	592 C > T	Ile198Phe	6	I	rs137852330	Southeast Asia	0.000	0.000	0.0001232	0.000	0.000003352	[234]
G6PD Viangchan	871 G > A	Gln291Lys	8	II	rs137852327	East Asian	0.000	0.001718	0.0004218	0.00006523	0.000	[235]
G6PD Kaiping	1388 G > A	Arg463His	12	II	rs72554664	Southeast Asian	0.000	0.003763	0.00007043	0.00002183	0.000001117	[234]

Allele frequency data for most variants are derived from gnomAD v4—Exomes, reported for African, East Asian, South Asian, American, and European populations. References correspond to primary studies linking these variants to malaria resistance. ^*^WHO classification of G6PD deficiency: Class I: severe deficiency (< 10% activity) with chronic non-spherocytic hemolytic anemia; Class II: severe deficiency (< 10%) without chronic hemolysis; Class III: moderate deficiency (10–60%), typically asymptomatic; Class IV: normal or very mild deficiency (> 60%), not clinically significant

The diverse spectrum of *G6PD* mutations exhibits pronounced geographic and population-specific patterns, reflecting localized adaptation shaped by long-term coevolution between humans and *Plasmodium*. In sub-Saharan Africa, the G6PD A- (202G > A and 376A > G) dominate with high frequency and concentrated distribution, indicative of low genetic heterogeneity. In contrast, Southeast Asian populations display high genetic diversity, with multiple coexisting variants such as G6PD Mahidol (487G > A), G6PD Viangchan (871G > A), and others, lacking a single predominant mutation [104]. This patternsuggests multiple independent selection events and more complex demographic histories in these regions. The ecological characteristics and transmission intensity of different *Plasmodium* species have played a critical role in shaping regional *G6PD* mutation profiles. *P.f* has predominantly driven the selection of the G6PD A- variants in Africa, whereas *P.v* is more likely responsible for the diversification observed in Southeast Asia. Additionally, historical factors such as human migration, genetic drift, and gene flow have further influenced the spread and maintenance of these mutations [105]. Altogether, G6PDd represents a compelling example of population-specific adaptation under pathogen-driven selection, serving as a genetic basis for malaria resistance, offering key insights into how the human genome responds to regionally heterogeneous selective pressures.

Similar to HbS, G6PDd is linked to a genetic mutation affecting RBCs. However, their effects on malaria infection are different. G6PDd reduces the risk of cerebral malaria but increases the risk of severe malarial anemia [106, 107]. Conversely, studies have confirmed that HbAS provides protection against both cerebral malaria and severe malarial anemia [18, 108]. The opposing effects of G6PDd and HbAS suggests that the mechanisms of malaria resistance in G6PDd and HbS are different, as parasite growth suppression alone does not fully explain their effects on malaria complications. Given the inconsistent selection effects of G6PDd and HbS, James A. Watson et al. proposed a balancing selection hypothesis, suggesting that the selective advantage arises from the competing risks of death from cerebral malaria and severe malarial anemia [109]. Another critical issue is the hemolysis caused by primaquine. Although G6PDd can reduce the risk of malaria infection, patients with G6PDd may still contract malaria. Primaquine was formerly one of the primary drugs used in the treatment of malaria, but it can cause hemolysis in individuals with G6PDd [110, 111]. Therefore, the widespread implementation of primaquine requires accurate diagnosis of G6PDd to minimize the risk of drug-induced hemolysis [112].

Given the crucial role of G6PD in the pentose phosphate pathway and clinical manifestations of G6PDd, most research has focused on oxidative stress in RBCs. Earlier studies have proposed that G6PDd enhances the oxidative state of RBCs, compromising membrane stability and thereby promoting parasite clearance from erythrocytes [113, 114]. Recent studies indicate that G6PD and pentose phosphate pathway also influence T cell activation [115, 116]. G6PDd may confer protection against cerebral malaria, potentially through modulation of T cell activation, which plays a central role in the disease’s pathogenesis [117]. Our previous study suggests that G6PDd mice show protection against experimental cerebral malaria due to a reduced T cell pro-inflammatory response [118]. These findings indicate that the mechanisms of malaria resistance in G6PDd differ from hemoglobinopathies, with G6PDd potentially affecting malaria pathology through immune regulation.

## Blood group system

**Fig. 6:**
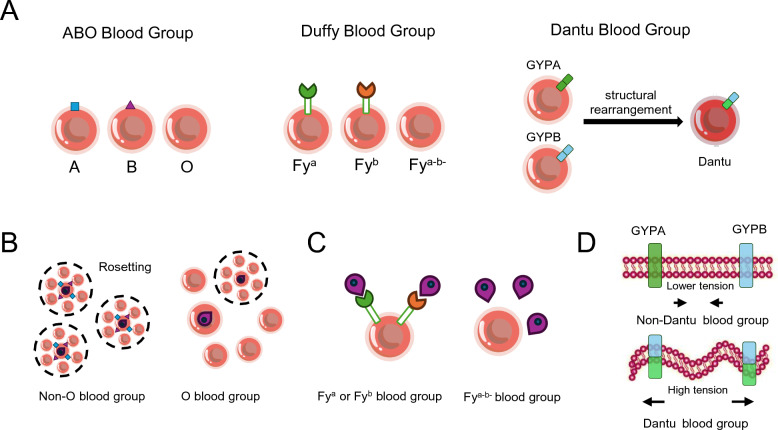
Blood group systems and mechanisms of malaria resistance. **A** Major blood group systems and molecular basis. The ABO blood group system is defined by the presence or absence of A and B antigens on the erythrocyte surface, determined by allelic variants of the ABO gene encoding glycosyltransferases. Blood group O lacks both A and B antigens due to an inactive transferase. The Duffy blood group system is determined by variants in the *FY* gene, encoding the Duffy antigen receptor for chemokines, producing primarily the Fy^a^ and Fy^b^ antigens. The Dantu blood group arises from a structural rearrangement between the glycophorin genes *GYPA* and *GYPB*, generating a hybrid gene encoding a chimeric sialoglycoprotein with distinct extracellular and intracellular domains. These blood group systems exhibit distinct genetic architectures and allele distributions across human populations. **B** Protective mechanism of the O blood group. Blood group O erythrocytes lack A and B antigens, resulting in reduced rosette formation, the adhesion of infected RBCs to uninfected erythrocytes. This decreases microvascular obstruction and parasite sequestration, mitigating the severity of *P.f* malaria. **C** Malaria resistance conferred by Duffy negativity. The Duffy-negative phenotype, characterized by the absence of Fy^a^ and Fy^b^ antigens on erythrocytes, is predominantly found in West and Central African populations. This phenotype results from genetic variants of the *FY* gene that abolish Duffy antigen expression on RBCs, thereby preventing *P.v* invasion. The near fixation of the Duffy-negative allele in these regions corresponds with the marked scarcity of *P.v* malaria, whereas Duffy-positive alleles remain prevalent in populations from Asia and the Americas where *P.v* transmission continues. **D** Malaria resistance mechanism associated with the Dantu blood group. The Dantu blood group results from a hybrid glycophorin gene formed by recombination between *GYPA* and *GYPB*. This structural alteration increases erythrocyte membrane tension, which impairs the ability of *P.f* merozoites to deform and invade RBCs

RBCs are a primary target for *Plasmodium* infection. Long-term natural selection has led to the preference of *Plasmodium* to different blood types. Several blood group systems have been confirmed to confer protective effects against malaria, including (a) The ABO blood group; (b) The Duffy blood group; (c) The Dantu blood group (Fig. 6A).

### ABO blood group

The ABO blood group system categorizes blood types based on the presence of specific antigens on RBCs: A, B, AB, and O [119]. Substantial evidence suggests that the origin, distribution, and relative proportions of human ABO blood types are influenced by selective genetic pressure from malaria parasite infection, particularly in malaria-endemic regions. Epidemiological studies have found that severe malaria is relatively more common in individuals with non-O antigens [120–122]. The current geographic distribution of the O group aligns with selective pressure favoring individuals with the O group in areas endemic for malignant malaria [123]. This phenomenon may be related to the influence of ABO genotypes on malaria parasite clustering, with the O blood type reducing the size and stability of malaria parasite-host RBC rosettes compared to non-O blood types [124] (Fig. 6B).

### Duffy blood group

The Duffy blood group system is based on the presence of Duffy antigens on the surface of RBCs, with the Duffy-negative phenotype being common in Africa [125] (Fig. 1F). This system primarily includes two antigens, Fy^a^ and Fy^b^, determined by the *FY* gene. *P.v* and *P.k* have been shown to invade RBCs through interactions with the Fy^a^ or Fy^b^ antigens of the Duffy blood group system [126–128]. The Duffy-negative phenotype is widely believed to confer resistance against *P.v* infection and is considered a potential target for malaria vaccines [129,130]. A study found that precursor cells from Duffy-negative individuals can transiently express Duffy antigens on their surface and support *P.v* infection, which may explain the low-density infections observed in various regions of Africa [131] (Fig. 6C). However, recent studies have shown that *P.v* can infect Duffy-negative individuals and has spread to regions with high Duffy negativity across Africa, suggesting an evolving invasion mechanism of *P.v* [132].

### Dantu blood group

The Dantu blood group is a low-frequency RBC antigen inherited in an autosomal dominant manner and is primarily found in East African populations. The molecular basis of the Dantu antigen is a structural rearrangement within the glycophorin gene cluster, specifically involving the glycophorin A (*GYPA*) and glycophorin B (*GYPB*) genes. This rearrangement produces two copies of a hybrid gene that encodes a chimeric sialoglycoprotein composed of the extracellular domain of GYPB and the intracellular domain of GYPA [133]. The altered structure interferes with the binding of parasite ligands such as erythrocyte-binding antigen-175 and erythrocyte-binding ligand-1, which are critical for merozoite invasion of RBCs [134].

Epidemiological studies have demonstrated that the Dantu blood group confers substantial protection against severe malaria, reducing the risk by approximately 30 ~ 40% [133, 135]. This protective effect is particularly pronounced in individuals homozygous for the Dantu allele, who exhibit a 74% reduction in the risk of severe *P.f* infection, comparable to the well-documented protection conferred by HbS [136].

The mechanism underlying this protection appears to involve increased RBC membrane tension, which inhibits parasite invasion and subsequent replication [135] (Fig. 6D). A controlled human malaria infection study confirmed that the Dantu blood group suppresses *Plasmodium* parasite growth in humans [137]. Although the frequency of the Dantu allele has increased in some regions of Kenya, it remains virtually absent in West Africa, highlighting its geographically restricted distribution likely due to region-specific selective pressures from malaria [133].

In the human blood group system, the O group [138], Duffy-negative [139], and Dantu blood group [140], all originate from pre-existing genetic polymorphisms, rather than de novo mutations that arose after the spread of malaria. These variants have been selectively enriched in specific populations due to their varying degrees of protective effects against malaria infection, resulting in pronounced patterns of population-specific adaptation. For instance, the Duffy-negative phenotype is nearly fixed in West African populations and serves as a major natural barrier to *P.v* invasion, while the Dantu variant is confined to certain East African populations and confers significant resistance to *P.f* invasion. The O blood group remains high in many malaria-endemic regions worldwide, particularly in sub-Saharan Africa, where its spatial distribution closely overlaps with areas of intense *P.f* transmission [123]. However, the frequency of O blood group is not universally elevated across all malaria-endemic regions [141], which may be attributed to adaptive trade-offs involving susceptibility to other infectious diseases such as cholera [142], and severe diarrheal illnesses [143, 144]. This pattern reflects the influence of balanced polymorphism. While malaria has been a major selective force, it is not the sole determinant of blood group distribution and variation.

## immune-related genes

The relationship between *Plasmodium* infection and the immune system is intricate and dynamic. Upon entering the body through a mosquito bite, the malaria parasite initially multiplies in liver cells before being released into the bloodstream to infect RBCs. This triggers a multi-level immune response from the host. The activated immune system aids in clearing the malaria infection, thereby limiting the parasite’s reproduction and spread. However, excessive immune responses can cause systemic inflammation and organ damage, such as cerebral malaria.

### CD36

The sequestration of *P.f*-infected RBCs in the brain microvasculature is a pathological hallmark of cerebral malaria. During malarial infection, brain microvascular endothelial cells become activated and express CD36 [145]. CD36 control host-parasite burden by regulating inflammatory responses [146]. CD36 has a high affinity to PfEMP1, which is expressed on the surface of parasitized RBCs, leading to the sequestration of the parasite [147]. Studies suggest that CD36 not only facilitates parasite sequestration but also plays a role in both innate and adaptive immunity in response to malarial infection [148]. A comparative study in Thailand also found that polymorphisms in the *CD36* gene promoter region (− 14 T > C and − 53G > T) and repeat sequences in intron 3 (12 TG repeats) are associated with the severity of cerebral malaria [149]. However, despite the observed associations between *CD36* polymorphisms and malaria severity, there remains limited evidence directly linking these genetic variations to malaria-driven selection. The high frequency of *CD36* mutations in African and Asian populations may have been shaped by selective pressures unrelated to malaria [150]. Therefore, while *CD36* remains a gene of interest in malaria pathophysiology, its evolutionary trajectory appears to be influenced by a complex interplay of environmental factors beyond malaria alone.

### Cytokines

Upon infection with *Plasmodium*, the host mounts a robust immune response, resulting in a cytokine storm that mediates both immune reaction and tissue damage [151]. Among the critical cytokines involved, IFN-γ and TNF-α play key roles in the pathogenesis [152, 153]. Several *IFNG* gene polymorphisms, including rs2069718, rs2069727, and (CA)n repeats, have been associated with susceptibility to severe malaria and parasite clearance across different populations [154, 155]. An early case–control study in African children reported a significant association between a promoter polymorphism in *IFNGR1* (− 56 T > C) and susceptibility to cerebral malaria [156].

Polymorphisms in the promoter region of the TNF gene, including − 238G > A, − 308G > A, − 857C > T, − 863C > A, and − 1031 T > C, have been shown to influence the expression levels of TNF-α in the host [157, 158]. Recent metal analysis has shown an association between − 308G > A and severe malaria caused by *P.f* [159]. A case–control study in Nigeria found a correlation between -238G > A and severe malaria [160]. Additionally, a comparative study in Thailand found that the frequencies of the C alleles at − 857C > T, − 863C > A, and − 1031 T > C were higher in patients with cerebral malaria than in patients with mild malaria [161]. The − 1031CC genotype has been associated with increased malaria episodes, while the − 308AA genotype correlates with protection, suggesting that TNF-α expression levels influence disease susceptibility and severity [162]. These findings highlight the dual nature of TNF-α in malaria pathogenesis, providing protective immune functions when appropriatelyregulated, but contributing to disease severity when overexpressed.

Interleukin-10 (IL-10) is an anti-inflammatory cytokine that mitigates tissue damage caused by the cytokine storm during malaria infection. A study in the Brazilian Amazon found that the C alleles of the − 592A > C and − 819 T > C polymorphisms are associated with decreased IL-10 levels and low parasite density [163]. A longitudinal prospective cohort study discovered that the − 1082A > G polymorphism is linked to decreased IL-10 levels and a reduced risk of clinical malaria in children [164]. Furthermore, a study in Kenyan children indicated that the 1082G/−819C/−592C (GCC) haplotype is associated with protection against severe malarial anemia and increased IL-10 levels [165]. The above findings present divergent perspectives. While differences in allele frequencies across populations may partially explain the relationship between IL-10 and malaria, further validation through alternative mechanisms in diverse populations is still required.

### CR1

Polymorphisms in the *CR1* gene primarily influence the density of complement receptor 1 (CR1) expression on RBCs, with certain variants leading to either high or low surface expression [166]. Reduced CR1 density on erythrocytes has been associated with an increased risk of severe malaria [167–169]. Along the Sino-Burmese border in Yunnan Province, the *CR1* variants 3093 T and 520 T, which are associated with reduced CR1 expression on erythrocytes, have been identified as region-specific polymorphisms linked to malaria prevalence [170]. In malaria-endemic regions such as Sardinia, a CR1 haplotype associated with low erythrocyte surface expression has been shown to be under positive selection, suggesting a potential evolutionary advantage in reducing malaria severity [171]. Consistently, genome-wide analyses have identified a novel *CR1* variant, rs12034598, under positive selection in other malaria-endemic populations [172]. While CR1 expression levels are strongly correlated with specific genotypes in non-African populations, including Europeans, South Asians, East Asians, and Oceanians, this genotype–phenotype correlation appears to be absent in African populations, possibly due to reduced activity of proteolytic enzymes that regulate CR1 cleavage and surface presentation [171].

CR1 has been identified as a key receptor for *P.f* invasion and plays multiple roles in the pathogenesis of malaria [173]. Specifically, CR1 facilitates the binding of *P.f*-infected RBCs to uninfected RBCs, leading to the formation of rosettes that can obstruct the microvasculature and exacerbate disease severity [174]. This rosetting process is primarily mediated by the PfEMP1, whose N-terminal segment (NTS-DBL1α) contains adhesive domains that interact directly with CR1 [175]. The *CR1* variant rs12034598 is associated with lower CR1 expression and a reduced erythrocyte sedimentation rate, both factors that may impair rosette formation, thereby limiting vascular obstruction and contributing to protection against severe disease [172]. Beyond its role in rosetting, CR1 also participates in the host immune response by mediating complement activation and facilitating the clearance of immune complexes [176].

### The immune interaction between the parasite and host

*P.f* has evolved multiple mechanisms to modulate host immunity and promote immune evasion. One such mechanism involves the secretion of a homolog of macrophage migration inhibitory factor, *P.f* macrophage migration inhibitory factor (PfMIF) during the blood stage, which alters monocyte/macrophage activation and suppresses cytokine production, thereby dampening inflammatory responses [177]. In addition, *P.f* induces epigenetic reprogramming of monocytes toward a regulatory phenotype, characterized by decreased expression of pro-inflammatory cytokines and reduced trimethylation of lysine 4 on histone H3 (H3K4me3) marks at inflammatory gene loci. This immunological shift blunts host immune responses upon reinfection and facilitates long-term parasite persistence [178]. *P.v* exhibits distinct immunomodulatory adaptations. It disrupts the balance between myeloid and plasmacytoid dendritic cells (DCs), impairs DC function, and enhances regulatory T cell (Treg) responses. Notably, the frequency and activation status of CD4⁺, CD25⁺ Tregs positively correlate with parasite density, suggesting that *P.v* actively promotes immune tolerance to support chronic infection [179, 180]. Moreover, in vitro studies have demonstrated that high ratios of *P.f*-infected RBCs can impair DC maturation in a dose-dependent manner. This is achieved by suppressing lipopolysaccharide (LPS)-induced co-stimulatory molecule expression and cytokine production, and in some cases triggering apoptosis. Importantly, these immunosuppressive effects occur independently of PfEMP1 and CD36, indicating the existence of an alternative, contact-independent mechanism of immune modulation [181]. Together, these findings highlight the multifaceted immune evasion strategies of *Plasmodium* species, which not only suppress initial host responses but also reprogram immune cells to facilitate parasite survival and long-term persistence.

## Others

### HMOX1

Heme is a major component of hemoglobin that can lead to the infiltration of inflammatory cells and tissue damage when free in the body. Conversely, free heme can contribute to resistance against severe malaria [182, 183]. Heme oxygenase-1 (HO-1), encoded by the *HMOX1* gene, is the rate-limiting enzyme in heme catabolism [184]. Polymorphisms in the (GT)(n) repeat of the *HMOX1* gene promoter region are associated with the expression levels of HMOX1 mRNA [185]. Previous studies found that shorter GT repeats correlate with lower HMOX1 expression and a higher risk of severe malaria [186]. However, a recent meta-analysis based on a large clinical dataset indicated that an increase in the length of the HMOX1 Short Tandem Repeat (STR) is unlikely to be associated with the occurrence of severe malaria [187].

### ATP2B4

The *ATP2B4* gene encodes the plasma membrane calcium ATPase 4 (PMCA4), the primary calcium pump that regulates intracellular Ca^2+^ homeostasis in RBCs during *Plasmodium* infection [188]. Genetic variation within an erythroid-specific DNase I hypersensitive site in an intron of *ATP2B4* has been identified as a key molecular basis affecting plasma membrane calcium ATPase isoform 4b (PMCA4b) expression and conferring innate resistance to malaria [189]. Multiple polymorphisms at the *ATP2B4* locus, including rs10900585, rs11240734, rs1541252, rs1541253, rs1541254, rs1541255, rs10751450, rs10751451 and rs10751452, with susceptibility to mild malaria, indicating that genetic variation at this locus may influence a broad range of malaria outcomes [190]. Population genetic analyses have revealed strong signatures of positive selection at the *ATP2B4* locus, particularly at rs10900588, in Northern Ugandan populations, providing compelling evidence of malaria-driven evolutionary pressure [191]. The protective *ATP2B4* genotype is prevalent in populations from The Gambia and other malaria-endemic regions, and is associated with significantly reduced peripheral parasitemia during infection [192]. Specifically, the rs10900585 variant confers a 64% reduction in the risk of placental *P.f* infection among homozygous primigravidae, highlighting the potential relevance of *ATP2B4* variation in pregnancy-associated malaria [193]. This protection is likely mediated through thedisruption of GATA binding protein 1 (GATA1)-regulated expression of an erythroid-specific transcript, leading to altered RBCs membrane properties and impaired parasite replication [194].

### NOS2

Nitric oxide (NO) is a critical mediator and signaling molecule in malaria immunopathology. Inducible nitric oxide synthase (NOS2) regulates NO levels under stress conditions. Polymorphisms in *NOS*, specifically − 954G/C, − 1173C/T, and the 2.6 kb CCTTT(n) microsatellite loci, have been significantly associated with the risk of *P.v* and *P.f* infections [195–197].

### PIEZO1

*PIEZO1* is a cation-selective mechanosensitive channel that regulates RBC volume, and gain-of-function variants can cause hereditary xerocytosis (HX), leading to abnormal RBC morphology [198]. Shang Ma et al. generated a mouse model of hereditary xerocytosis carrying a *PIEZO1* mutation, thereby demonstrating that *PIEZO1* mutation impair early *Plasmodium* growth and confer resistance to experimental cerebral malaria [199]. Recently, it was confirmed that the E756del mutation is strongly associated with protection against severe malaria in heterozygotes [200]. Furthermore, Christian N. et al. identified an epistatic interaction between *PIEZO1* E756del and HbS in Gabonese children, whereby the *PIEZO1* variant reduced PfEMP-1 surface expression without affecting RBCs hydration or parasite growth [200]. RBCs mediate protection against *Plasmodium* infection and cerebral malaria through gain-of-function *PIEZO1*, while T cells also contribute to the prevention of cerebral complications [199].

In addition to well-characterized genes, several studies conducted in genetically and geographically distinct populations have uncovered additional loci associated with severe malaria. However, due to limited population specificity, these genes are not listed individually in earlier sections. For example, a study in Kilifi County, Kenya, identified significant associations between severe malaria and polymorphisms in 15 genes or loci, most of which are involved in RBC biology [201]. Similarly, a case–control study in Tanzania implicated variants in *USP38*, *FREM3*, *SDC1*, *DDC*, and LOC727982 as potential contributors to disease susceptibility [202]. Moreover, a genome-wide association study (GWAS) identified regulatory variants rs116525449 and rs79644959, which modulate ARL14 expression in immune cells and influence antigen presentation to T lymphocytes, thereby contributing to the development of severe malaria [203]. More recently, research in malaria-endemic populations in Cameroon revealed novel loci such as *CHST15* and *SOD2*, with *SOD2* demonstrating particularly strong protective effects during the early stages of *Plasmodium* infection [204].

## Epigenetic regulation and malaria susceptibility

Emerging evidence highlights the pivotal role of epigenetic mechanisms, such as DNA methylation, histone modifications, chromatin remodeling, and non-coding RNAs, in regulating parasite gene expression and modulating host immune responses during *Plasmodium* infection [205]. Previous studies have demonstrated that histone deacetylases (HDACs) and histone acetyltransferases (HATs) coordinately modulate transcriptional plasticity across different developmental stages of the parasite [206]. For instance, the HDAC inhibitor apicidin induces global histone hyperacetylation and concomitant downregulation of activating histone marks such as H3K4me3, leading to transcriptional dysregulation and impaired parasite development [207]*.*

Although evidence for host epigenetic regulation during malaria infection remains limited, emerging studies suggest its involvement in shaping infection outcomes. LaMonte et al. reported that erythrocyte-derived miRNAs, specifically miR-451 and let-7i enriched in HbS RBCs, are translocated into *P.f* where they bind parasite mRNAs and suppress translation, indicating an intrinsic epigenetic defense mechanism mediated by host miRNAs [208]. Additionally, *P.v* infection has been shown to alter miRNA expression profiles associated with erythropoiesis in erythroid progenitor cells [209]. In a *Plasmodium chabaudi* blood-stage infection model, vaccine-protected BALB/c mice exhibited liver-specific miRNA reprogramming during crisis, which was associated with spontaneous resolution of infection [210]. However, some studies have indicated that for certain candidate genes (such as *PKLR* gene), although regulatory region variants are present, the antimalarial mechanism is more likely attributable to functional mutations in the coding region rather than epigenetic regulation [211]. Currently, most studies on host epigenetics are restricted to specific populations or conditions, underscoring the need for large-scale, population-diverse validation studies [212].

Epigenetic modifications also play a central role in modulating immune responses during malaria infection. For example, H3K4me3 is associated with enhanced inflammatory responses in monocytes and natural killer cells upon re-infection [213]. In Ugandan children, elevated levels of H3K27 methylation and arginine dimethylation, along with reduced expression of histone variant H3.3 in monocytes, were linked to suppressed pro-inflammatory cytokine responses. This epigenetic signature correlated with asymptomatic parasitemia and predicted the future acquisition of naturally acquired clinical immunity [214]. Furthermore, a longitudinal study in Burkinabe children revealed hypomethylation at CpG sites within the promoter region of the *TNF* gene during symptomatic malaria episodes, which was associated with increased TNF expression, highlighting the regulatory role of DNA methylation in pro-inflammatory gene activation [215].

Collectively, these findings suggest that both host and parasite epigenetic mechanisms contribute significantly to the initiation, regulation, and outcome of malaria infection. In particular, epigenetic regulation offers a complementary layer of explanation beyond genomic variation for the control of parasite development and host immune responses. Future research should focus on functional validation in human clinical samples and explore the translational potential of targeting epigenetic regulators for malaria control strategies.

## Technological advancements in malaria susceptibility research

GWAS and CRISPR-based functional genomics have greatly accelerated the discovery and validation of human genetic variants conferring resistance to malaria. GWAS systematically scan the genome to identify common genetic variants associated with disease susceptibility or resistance across large populations. GWAS, particularly those conducted in diverse African populations, have expanded the landscape of candidate loci beyond classical hemoglobinopathies. For example, variants near *ATP2B4* [216], *PTPRT*, *MYLK4*, *VENTX*, *UROC1*, and *ACER3* [217] have been linked to differential susceptibility to severe malaria. These findings emphasize the polygenic and population-specific nature of malaria resistance and the need for broader representation of endemic regions in genomic research.

CRISPR-Cas9-based functional genomics enables precise editing of specific genes to experimentally determine their roles in biological processes, allowing direct validation of candidate variants uncovered by genomic studies. CRISPR interference (CRISPRi) screens have revealed host factors essential for *P.f* invasion and replication in hepatocytes and erythrocytes, as well as genes involved in modulating the hostresponse to drug-resistant parasite strains [218]. Moreover, in vivo CRISPR-engineered animal models, including mice carrying humanized RBCs or specific allelic variants such as PIEZO1 gain-of-function mutations-have provided critical insights into the role of host genes in modulating vascular pathology and cerebral malaria outcomes [219]. These in vivo systems capture complex immune responses and tissue-specific effects that are difficult to replicate in vitro, thereby enhancing the translational relevance of functional findings.

Together, these genomic and functional approaches form a synergistic pipeline, enabling the identification of resistance loci at the population level and subsequent validation of their biological roles.

## Conclusion

In conclusion, human genetic variations continue to provide invaluable insights into the complex evolutionary arms race between *Plasmodium* parasites and their hosts. The dynamic interplay between host and parasite reflects ongoing natural selection on both sides: while human populations have evolved protective variants such as HbS, HbC, G6PDd, Duffy negativity, α- and β-thalassemia, and structural variants like Dantu, *Plasmodium* species have simultaneously adapted through mechanisms such as antigenic variation, immune modulation, and drug resistance. Notably, multiple protective mutations converge on RBC structure and function, disrupting parasite entry, growth, or cytoadherence—highlighting the erythrocyte as a central battlefield in host-parasite coevolution. Furthermore, variants in immune regulatory genes suggest that the host inflammatory response is another key target of evolutionary pressure.

However, Current research on human genetic resistance to malaria has provided valuable insights but remains limited by several important factors. First, most genomic studies have disproportionately focused on specific populations, particularly in sub-Saharan Africa, leaving regions such as Southeast Asia, Oceania, and South America underrepresented. This limits our understanding of population-specific variants and may overlook important genetic adaptations shaped by different *Plasmodium* species or environmental pressures. Second, many association studies lack sufficient sample sizes or longitudinal follow-up, which reduces statistical power and makes it difficult to establish causality or assess the long-term clinical implications of candidate variants. Third, despite the identification of numerous genetic loci associated with malaria outcomes, functional validation of these variants remains incomplete, often relying on in vitro systems that do not fully capture the complexity of host-parasite interactions in vivo.

To address these limitations, future strategies should prioritize the inclusion of genetically diverse and geographically varied populations in genomic research, supported by harmonized phenotyping protocols and comprehensive clinical data. Functional validation must also be expanded using CRISPR-based screens, high-throughput functional assays, and physiologically relevant models such as humanized mice and ex vivo organoid cultures. Integrating GWAS findings with epigenomic, proteomic, and microbiome data may uncover novel regulatory networks and host–pathogen dynamics. Additionally, understanding how genetic variants interact with evolving parasite strains, environmental factors, and public health interventions will be essential for developing context-specific and equitable malaria control strategies.

## Supplementary Information

Supplementary material 1.

## Data Availability

Not applicable.

## Abbreviations

Cas9: CRISPR-associated protein 9
CR1: Complement receptor 1
CRISPR: Clustered regularly interspaced short palindromic repeats
DC: Dendritic cell
G6PD: Glucose-6-phosphate dehydrogenase
G6PDd: Glucose-6-phosphate dehydrogenase deficiency
GWAS: Genome-Wide association study
H3K4me3: Trimethylation of lysine 4 on histone H3
Hb: Hemoglobin
HbA: Hemoglobin A
HBB: Hemoglobin subunit beta
HbC: Hemoglobin C
HbE: Hemoglobin E
HbF: Fetal hemoglobin
HbH: Hemoglobin H
HbS: Hemoglobin S
HDAC: Histone deacetylase
IFN-γ: Interferon γ
IgG: Immunoglobulin G
IL-10: Interleukin-10
*P.f*: *Plasmodium falciparum*
PfEMP1: Erythrocyte membrane protein 1
PIEZO1: Piezo-Type Mechanosensitive Ion channel component 1
*P.k*: *Plasmodium knowlesi*
*P.m*: *Plasmodium malariae*
*P.o*: *Plasmodium ovale*
*P.v*: *Plasmodium vivax*
RBC: Red blood cell
SCD: Sickle cell disease
TNF-α: Tumor necrosis factor-α
ZnPP: Zinc protoporphyrin IX
