# Supplementary material for: Human genetic variations conferring resistance to malaria
Source: J Transl Med. 2025 Sep 24;23:997. doi: 10.1186/s12967-025-07017-w (PMC12462182; doi:10.1186/s12967-025-07017-w)
Supplement: Supplementary file 1 — Supplementary material 1. [file 12967_2025_7017_MOESM1_ESM.pdf]

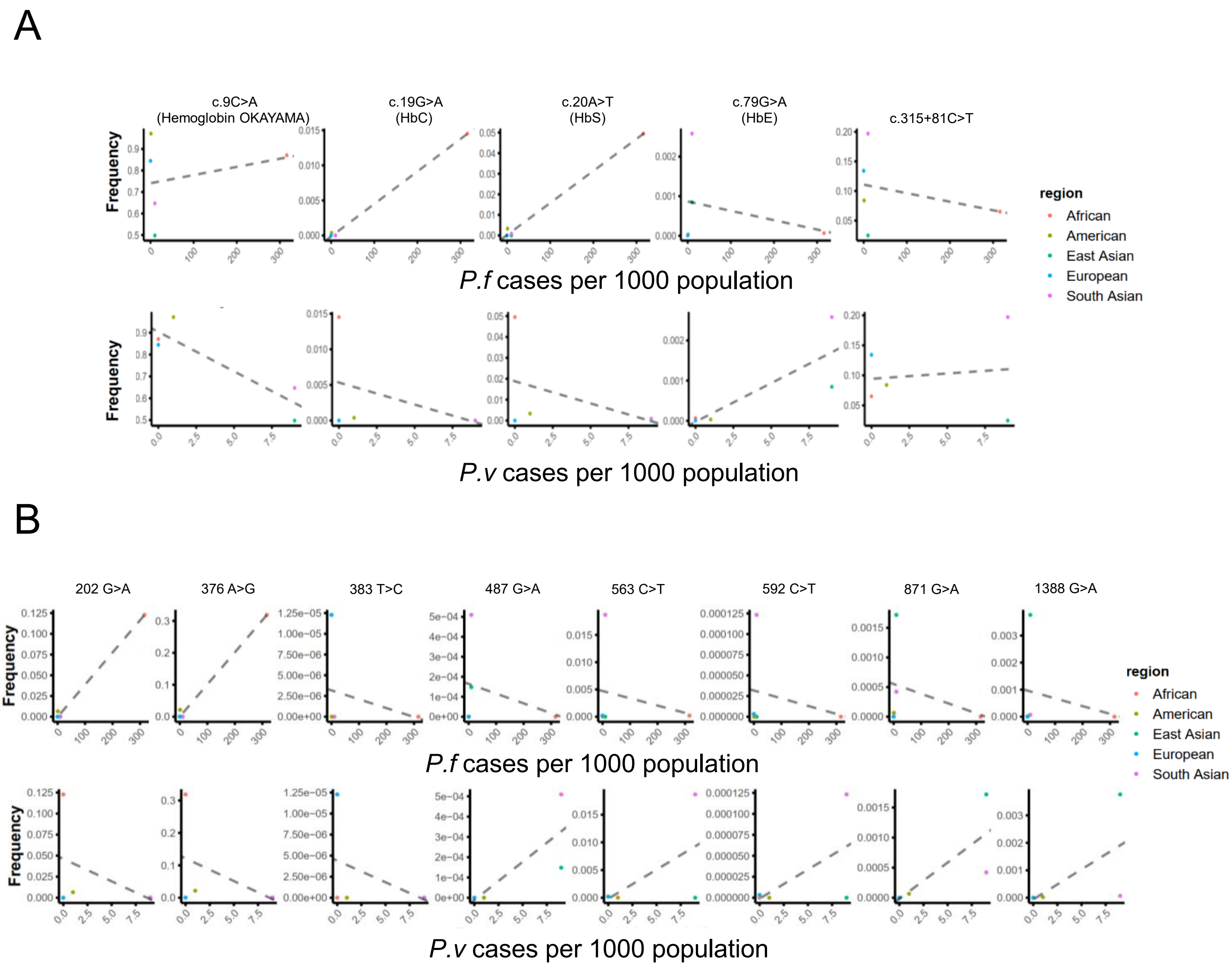

Supplement Figure S1

Regional Distribution and Correlation of Malaria Burden with Human Genetic Variant Frequencies

Each subplot displays the relationship between the regional incidence of *Plasmodium falciparum* (top row) or *P. vivax* (bottom row) infections per 1,000 population (x-axis) and the frequency of a specific human genetic variant (y-axis). Each point corresponds to a distinct geographic region, color-coded by region identity. Dashed lines represent linear regression models fitted to the data within each panel. Malaria incidence data are derived from the World Malaria Report and reflect 2020 case estimates. Allele frequency data are obtained from the gnomAD v4 – Exomes dataset; the frequency of variant c.315+81C>T is sourced from the 1000 Genomes Project.
